# Synthesis and Matrix Properties of α-Cyano-5-phenyl-2,4-pentadienic Acid (CPPA) for Intact Proteins Analysis by Matrix-Assisted Laser Desorption/Ionization Mass Spectrometry

**DOI:** 10.3390/molecules25246054

**Published:** 2020-12-21

**Authors:** Antonio Monopoli, Angelo Nacci, Tommaso R. I. Cataldi, Cosima D. Calvano

**Affiliations:** 1 Agenzia delle Dogane e dei Monopoli, Ufficio delle Dogane di Bari, Corso De Tullio, 70122 Bari, Italy; antomono@libero.it; 2 Dipartimento di Chimica, Università degli Studi di Bari Aldo Moro, Via Orabona, 70126 Bari, Italy; angelo.nacci@uniba.it (A.N.); tommaso.cataldi@uniba.it (T.R.I.C.); 3 Centro Interdipartimentale di Ricerca S.M.A.R.T., 70126 Bari, Italy; 4 Dipartimento di Farmacia-Scienze del Farmaco, Università degli Studi di Bari Aldo Moro, Via Orabona, 70126 Bari, Italy

**Keywords:** MALDI matrix, mass spectrometry, proteomics, bacteria, hazelnut, milk

## Abstract

The effectiveness of a synthesized matrix, α-cyano-5-phenyl-2,4-pentadienic acid (CPPA), for protein analysis by matrix-assisted laser desorption/ionization time-of-flight mass spectrometry (MALDI-TOF MS) in complex samples such as foodstuff and bacterial extracts, is demonstrated. Ultraviolet (UV) absorption along with laser desorption/ionization mass spectrometry (LDI-MS) experiments were systematically conducted in positive ion mode under standard Nd:YLF laser excitation with the aim of characterizing the matrix in terms of wavelength absorption and proton affinity. Besides, the results for standard proteins revealed that CPPA significantly enhanced the protein signals, reduced the spot-to-spot variability and increased the spot homogeneity. The CPPA matrix was successful employed to investigate intact microorganisms, milk and seed extracts for protein profiling. Compared to conventional matrices such as sinapinic acid (SA), α-cyano-4-hydroxycinnamic acid (CHCA) and 4-chloro-α-cyanocinnamic acid (CClCA), CPPA exhibited better signal-to-noise (S/N) ratios and a uniform response for most examined proteins occurring in milk, hazelnut and in intact bacterial cells of *E. coli*. These findings not only provide a reactive proton transfer MALDI matrix with excellent reproducibility and sensitivity, but also contribute to extending the battery of useful matrices for intact protein analysis.

## 1. Introduction

In the last three decades, matrix-assisted laser desorption/ionization time-of-flight mass spectrometry (MALDI-TOF MS) [1] has become a suitable technique to analyse large biomolecules such as oligonucleotides, peptides and proteins in complex samples [2]. Proteomics strategies have been predominantly developed in clinical, food and microbiological fields for biomarker discovery [3,4], food authentication [5,6], control of the technological processes [7], bacterial species identification [8,9], etc. These approaches are based on the acquisition of protein “fingerprints” directly from biological fluids [10], foodstuffs or intact microorganisms relating two or more inherent features (e.g., healthy/diseased, authentic/adulterated, Gram-positive/Gram-negative, etc.) [11]. Compared to DNA-based protocols [12,13], protein fingerprinting by MALDI-MS was demonstrated to be easier, cheaper, faster and more reliable. For instance, the identification of bacteria and fungi for diagnostic purposes is currently achieved by MALDI-MS profiling due to its high resolution and low cost for single determination making it an effective alternative to false negative/positive biochemical tests [14]. MALDI-MS is considered a valuable tool in routine clinical microbiology laboratories for rapid and cost-effective species identification, either directly from cultures or from clinical specimens such as plasma, blood, and urine or for the recognition of antibiotic resistance and bacterial typing [15]. Moreover, this technique has been included in conventional proteomic strategies applied to food analysis [16] with the aim of safety and quality control in identifying adulteration, authentication, occurrence of hidden allergens and so on [17,18,19].

Despite the large use of MALDI-MS in many analytical areas, some major drawbacks remain; for instance, the variability of signal intensities and resolution between different spots of the same sample. Furthermore, the resulting inhomogeneous crystallization is one of the major factors limiting the application of MALDI-MS for quantification and for spatially resolved imaging [20]. Numerous efforts have been ended to overcome these limitations of MALDI-MS, such as the use of ionic liquids to improve spot homogeneity [21,22], the exploration of novel matrices able to produce few interfering background signals in the analysis of low-molecular-weight analytes [23,24,25,26] and the application of strong base matrices as proton sponges [27] or an electron transfer matrix [28] to facilitate the ionization of nonpolar compounds [29,30]. While several alternative approaches have been proposed to obtain better performances in terms of reproducibility, resolution, sensitivity and dynamic mass range of low-mass compounds, the use in proteomic MALDI-MS investigations of sinapinic acid (SA) and α-cyano-4-hydroxycinnamic acid (CHCA), either in dried-droplet or surface preparation mode, still constitutes the ‘gold standard’. Moreover, the whole bacterial cell analysis by CHCA matrices gives rise to a homogeneous sample/matrix spot on the target compared to “hot spots” formation by SA, which leads to shot-to-shot and spot-to-spot lack of reproducibility [31]. A main drawback of CHCA is represented by its marked preference for strongly basic arginine-containing peptides [32] and the attendant suppression of acidic peptides, limiting the sequence coverage and thus hindering the identification of low-abundance proteins. Indeed, a rationally designed chloro-cinnamic derivative of CHCA, namely 4-chloro-α-cyanocinnamic acid (CClCA), was introduced to overcome this issue, demonstrating better performances in ionizing acidic peptides thanks to its lower proton affinity [32]. Therefore, the search for a matrix exhibiting a uniform response for intact proteins of different basicity and weight is still demanded.

In this work, α-cyano-5-phenyl-2,4-pentadienic acid (CPPA) was synthesized, characterized by laser desorption ionisation (LDI) mass spectrometry and tested as a MALDI matrix for large biomolecule analysis. CPPA performed very successfully when employed as a matrix for intact proteins, exhibiting satisfactory signal-to-noise ratios compared to SA, CHCA and CClCA and an improved intra-spot and spot-to-spot repeatability. Higher sensitivity at a lower laser energy was also ascertained, leading to good resolution and reduced fragmentation.

## 2. Results and Discussion

### 2.1. Synthesis and Characterisation of CPPA

The targeted CPPA matrix was synthesized by following the standard Knoevenagel condensation reaction using cyanoacetic acid and cinnamaldehyde [33]. Upon the chemical synthesis and ensuing sample purification by repeated recrystallization, CPPA was examined by ^1^H-NMR by dissolving it in DMSO-*d*_6_; the acquired spectrum was in good agreement with previous literature data [34].

**Figure 1:**
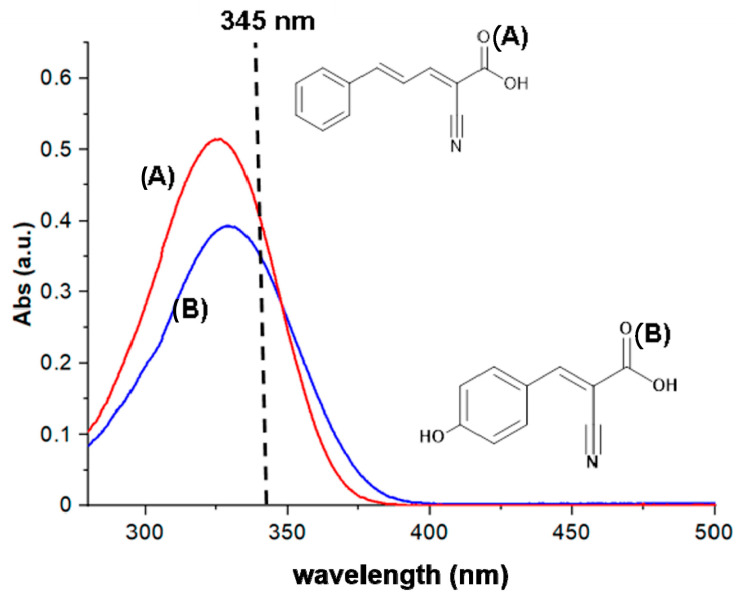
UV absorption spectra of (**A**) CPPA and (**B**) CHCA with relevant molecular structures. The laser wavelength used in MALDI-MS at 345 nm is marked with a vertical dotted line. (**A**) CPPA: α-cyano-5-phenyl-2,4-pentadienic acid; (**B**) CHCA: α-cyano-4-hydroxycinnamic acid.

Regarding the UV spectrum of CPPA in acetonitrile/water mixture, 2:1 (v:v), a good absorption efficiency at the laser excitation wavelength of 345 nm was evidenced (Figure 1), which seems comparable to that of CHCA. Indeed, CPPA exhibits a relatively strong absorption at the applied laser wavelength with a molar extinction coefficient of ε = 22,500 L/mol·cm, which is roughly equivalent to that of CHCA (ε = 20,200 L/mol·cm) evaluated in the same experimental conditions. Note that the extinction coefficients of CPPA and CHCA were evaluated at 345 nm even though other Nd:YLF lasers stated an emission wavelength at 349 or 351 nm. For the sake of comparison, we also estimated the ε values at both these last wavelengths; while at 349 nm, the same value (ε = 17,530 L/mol·cm) for CPPA and CHCA was obtained, a higher extinction coefficient for CPPA at 351 nm was estimated, i.e., ε_CPPA_ = 16,700 vs ε_CHCA_ = 15,400 L/mol·cm. A similar trend was reported by Bahr and Jaskolla [35], who calculated ε values at different wavelengths even if the results are not directly comparable to our estimations due to diverse experimental conditions employed, such as matrix concentration and solvent used [36]. Apparently, the extinction coefficients of CPPA at 345 nm CPPA meets one of the basic requirements of being a good UV-absorption MALDI matrix. Note that a previous study demonstrated that CPPA acts as a strong absorbing organic chromophore in addition to liquid matrices for the analysis of polyglycols and poly(dimethylsiloxane) [37]. Here, we demonstrated that CPPA can work as a good performing matrix itself for the analysis of intact proteins (vide infra). Therefore, its behaviour was investigated by laser desorption ionisation (LDI) MS experiments.

**Figure 2:**
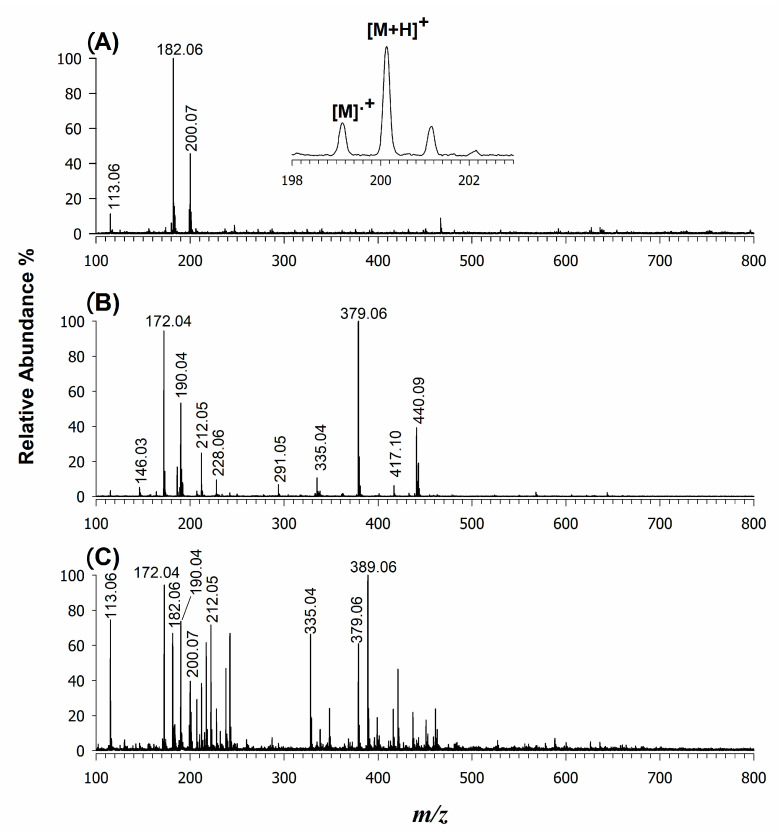
Positive LDI-MS spectra of matrices: (**A**) CPPA with a fluence of 3 mJ/cm^2^, (**B**) CHCA at 10 mJ/cm^2^ and (**C**) an equimolar mixture of CPPA and CHCA (5 mJ/cm^2^). LDI-MS: laser desorption/ionization mass spectrometry.

Figure 2A shows the LDI mass spectrum of CPPA in positive ion mode in the range 100–800 *m*/*z*. Three main peaks were observed in the MS spectrum, namely at *m*/*z* 200.07, assigned to a protonated adduct ([M + H]^+^), where M represents the neutral compound; at *m*/*z* 182.06, corresponding to a species having lost a water molecule ([M − H_2_O + H]^+^); and at *m*/*z* 113.06, likely ascribed to a 2,4-hexa-2,4-dienoyl-oxonium product ion with the raw formula [C_6_H_9_O_2_]^+^. The low background signals of CPPA (Figure 2A) can be attributed to the lower laser fluence (ca 3 mJ/cm^2^ for CPPA vs. 10 mJ/cm^2^ for CHCA) required for the ionization/desorption processes, which reduce the energy of intermolecular collisions, thus limiting the adduct formation [38,39,40]. Figure 2B shows the LDI mass spectrum of the CHCA matrix using the same experimental conditions. As evident, the spectrum reveals a consistent number of peaks besides the expected protonated adduct at *m*/*z* 190.05; these peaks were generated from complex structures identified as dimers and trimers also including sodium and potassium adducts [41,42]. An additional series of experiments was performed by mixing different molar ratios of CHCA and CPPA followed by LDI-MS. As an example, the LDI mass spectrum of Figure 2C was acquired with an equimolar ratio of CHCA and CPPA. Looking at the protonated adducts of both matrices, it is evident that the mass spectrum is not dominated by CHCA or CPPA peak signals, suggesting a possible mutual proton transfer. However, both the signals related to CHCA ([M + H]^+^ at *m*/*z* 190.04 and [M − H_2_O + H]^+^ at *m*/*z* 172.04) showed a slightly higher intensity compared to the same corresponding ions of CPPA (i.e., [M + H]^+^ at *m*/*z* 200.07 and [M − H_2_O + H]^+^ at *m*/*z* 182.06), thus suggesting a favoured proton transfer to CHCA related to differences in proton affinities. Using density functional theory (DFTB3) calculations, the theoretical proton affinity (PA) of both CPPA and CHCA were estimated to be 841 ± 3 kJ/mol and 866 ± 3 kJ/mol, respectively; the PA value of CHCA is in good agreement with previous data of Mirabelli and Zenobi [43] estimated by a B3LYP/6-311++G(3df,3pd) function. It is expected that in both cases, the positive charge of protonated matrices initially located either at the carboxylic or at the cyano residue is delocalized over the entire conjugated π-electron system; however, the electron-donating effect of the 4-OH group in CHCA can stabilize the protonated CHCA matrix molecule more, resulting in a slightly larger PA than CPPA, where the charge delocalization is much less pronounced due to the absence of the functional group in the para position. Conceivably, we speculate that a more efficient proton transfer process from CPPA matrix ions to analytes might occur because of its PA resulting in a more uniform protonation of low-basicity compounds (vide infra). Indeed, a low PA corresponds to a high protonation power of the [M + H]^+^ matrix ion that allows a better proton transfer with consequently more sensitive analyte detection. The minimum requirements for a compound to behave as a matrix was investigated here, but of course, many other critical factors should be considered to explain primary ion formation from the matrix (M) and secondary ion formation of the analyte (A) [44]. For instance, the observation of a small contribution of the odd-electron molecular ion M^•+^ of CPPA at *m*/*z* 199.06 (see inset in Figure 2A) suggests the occurrence of a multiple photon absorption or an “energy pooling” mechanism [43] that is either difficult to invoke or rule out, since the ionization energy of CPPA is not available. Another likely mechanism that can be considered is the electronic excitation proton transfer (ESPT), where only one photon is required, that generally results in an increase of the matrix acidity, promoting the proton transfer to the analyte [45]. Since ESPT does not affect carboxylic acids [46], it cannot be invoked as an effective mechanism for the proton transfer of CPPA. Further, the plume processes can be examined from both a thermodynamic and a kinetic point of view, but without having a special instrument setup, such as a split sample probe [43], any data attained should be expected as the result of both primary and secondary ionization processes.

### 2.2. CPPA as a MALDI Matrix of Standard Proteins

**Figure 3:**
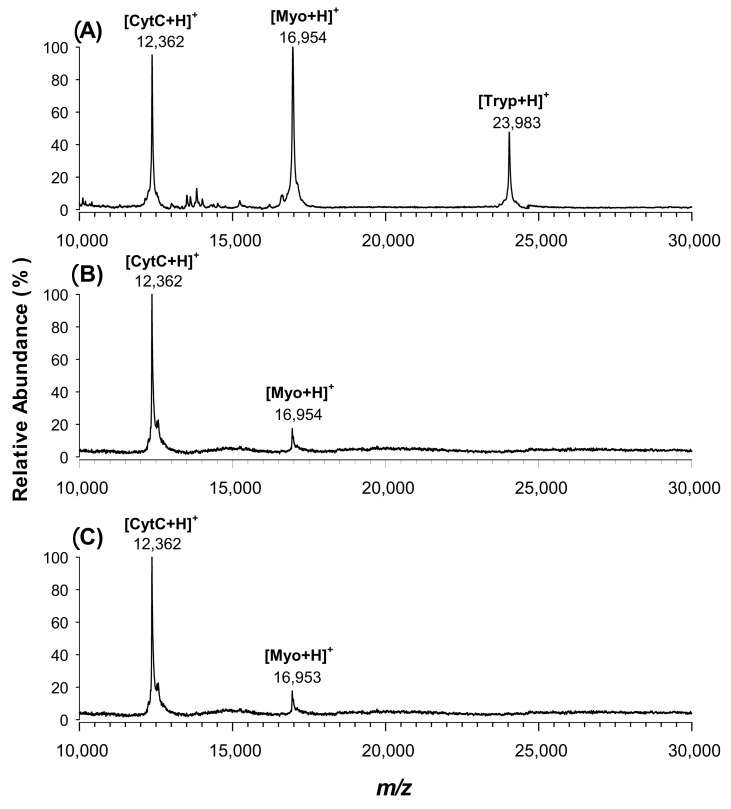
Positive MALDI-TOF MS spectra of a standard equimolar (100 fmol/µL) mix of cytochrome *c* (cytC), bovine myoglobin (Myo) and trypsinogen (Tryp) using (**A**) CPPA, (**B**) CHCA or (**C**) CClCA as the matrix.

**Figure 4:**
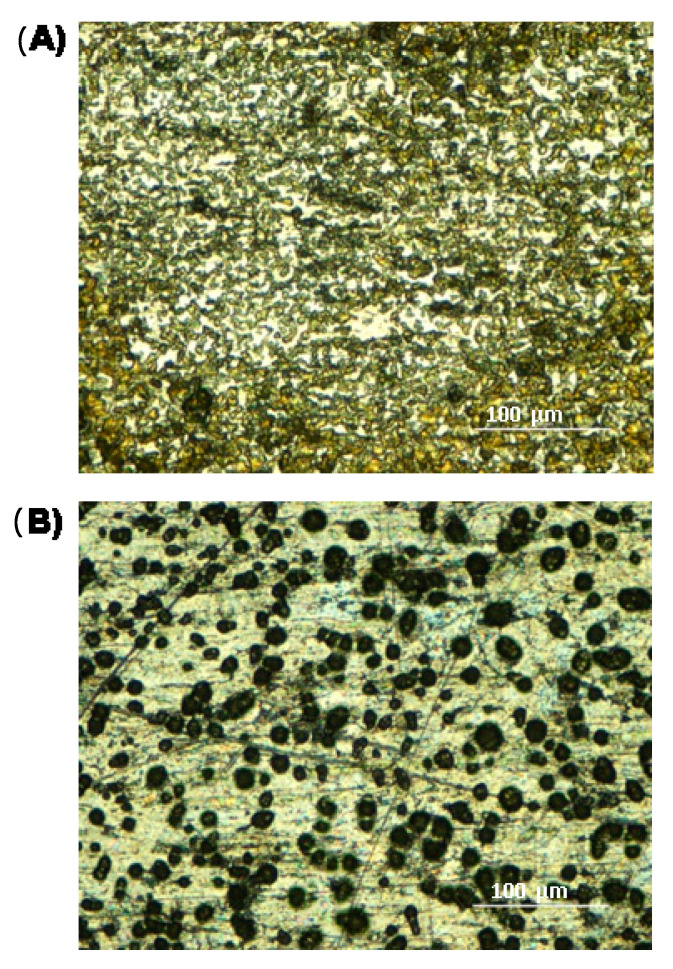
Microscope images of (**A**) CPPA and (**B**) CHCA matrices after mixing with the standard protein solution.

Once CPPA was chemically characterized, its capability as a MALDI matrix was examined using a standard protein mixture (100 fmol/µL of each) of cytochrome *c* (12,361 Da), bovine myoglobin (16,953 Da) and trypsinogen (23,981 Da), and results were compared to SA, CClCA and CHCA matrices. In the relevant spectrum of Figure 3A, three main peaks at *m*/*z* 12,362, 16,954 and 23,983 were easily detected, corresponding to the protonated adducts of each target protein, respectively. CPPA promoted monomeric singly charged ions to a high degree, leading to uniform data; the absolute intensity of protein peaks was relatively lower when using CHCA (Figure 3B) and CClCA (Figure 3C), where the lower-molecular-weight proteins cytochrome *c* and bovine myoglobin were easily ionised, while the trypsinogen ion was not at all detected at 100 fmol/µL concentration. Typically, the spectral quality obtained using CPPA in replicate analyses was better than that obtained using CHCA in terms of signal-to-noise (S/N) ratio and threshold laser fluences. While CPPA provided a ca. threefold improvement in the S/N ratio in comparison to CHCA, a twenty-fold higher value was obtained compared to SA (data not shown). These better values of the S/N ratios may be related to a different crystallization behaviour, since a more homogenous spot is formed using the CPPA matrix. The optical microscope images of CPPA and CHCA at 100× magnification are reported in Figure 4A,B, respectively. A thin and homogeneous layer of the matrix was found on the target plate covered with CPPA (Figure 4A), while small crystals evenly homogeneously distributed all over the matrix spot were discernible with CHCA (Figure 4B). The crystal size inspected microscopically was larger in CHCA compared with CPPA, which is in agreement with the fact that a benefit is achieved for this last matrix over the normal preparation of dried droplets that was found to be the simplest and most effective deposition technique and was thoroughly used in this work.

### 2.3. CPPA as a Matrix for Complex Samples by MALDI-TOF MS

Once the matrix was successfully tested on standard purified proteins, we applied the CPPA matrix to the analysis of more complex protein-containing samples, including milk and hazelnuts. Milk proteins are key components providing essential amino acids vital for energy, tissue growth and cellular function, also acting as hormones or displaying antimicrobial properties. A promising outcome of milk proteome analysis is the discovery of biomarkers of disease conditions, thus enabling farmers and veterinarians to easily screen diseases and suggest novel treatment options. In dairy science, proteomics is also largely used to characterize low-abundance proteins to monitor the changes in protein composition during storage or processing, to identify adulteration in high-quality milk, to discriminate milk source for cheese manufacturing or to recognize allergic proteins [47]. On average, cow’s milk contains approximately 3–4% of protein, even if this level can fluctuate with breed, primary source of nutrition, stage of lactation and health and dietary status of the animal [48]. Caseins represent approximately 80% of total bovine milk and approximately 18% of whey proteins [48,49]. Five types of casein (CN) are present in bovine milk: α-S1-CN, α-S2-CN, β-CN, k-CN and ϒ-CN; the latter being breakdown products cleaved from β-CN by the proteolytic plasmin enzyme [42]; they are found usually in the following relative content: α-S1-CN > β-CN > k-CN > α-S2-CN. The most abundant whey proteins are β-lactoglobulin (β-LG) and α-lactalbumin (α-LA), which represent approximately 60% and 20% of total whey proteins, respectively, followed by a small amount of bovine serum albumin (BSA) and immunoglobulins (Igs).

**Figure 5:**
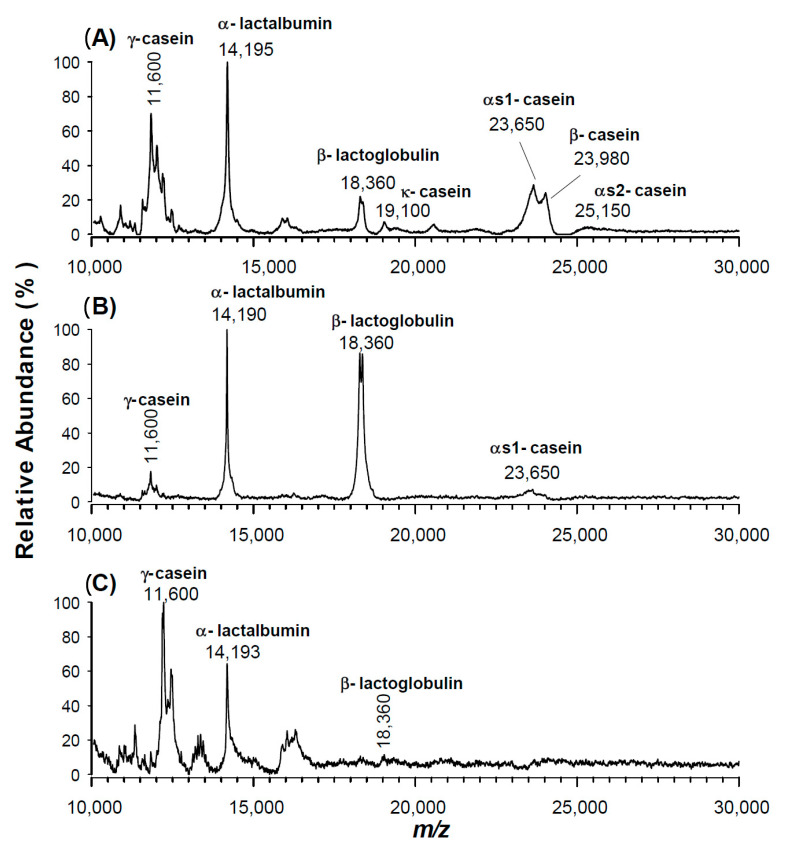
Positive MALDI-TOF MS spectra of an aqueous extract of cow milk proteins after mixing with (**A**) CPPA, (**B**) CHCA or (**C**) CClCA as the matrix. The identified proteins are labelled in the subfigures.

Here, bovine milk samples were simply diluted with water and analysed without any further purification by using CPPA in comparison with other conventional matrices. In Figure 5, the MALDI mass spectrum obtained in the same experimental conditions using CPPA, CHCA or CClCA are compared. All milk proteins were observed as protonated adducts when CPPA was employed as a matrix: ϒ-casein (*m*/*z* 11,600), α-lactalbumin (*m*/*z* 14,195), β-lactoglobulin (*m*/*z* 18,360), k-casein (*m*/*z* 19,100), α-S1-casein (*m*/*z* 236–50), β-casein (*m*/*z* 23,980) and α-S2-casein (*m*/*z* 25,150). Only whey proteins were promptly detected with the CHCA, CClCA or SA matrix (data not shown); furthermore, a small signal of α-S1-casein was detected using CHCA (Figure 5B), while the other caseins were greatly suppressed because of their high degree of phosphorylation that limits the ionization efficiency of conventional matrices [50]. Conversely, ϒ-casein at *m*/*z* 11,600 Da was observed by employing CClCA (Figure 5C), also suggesting a preferential ionization by this matrix to lower molecular weight (MW) proteins or polypeptides.

**Figure 6:**
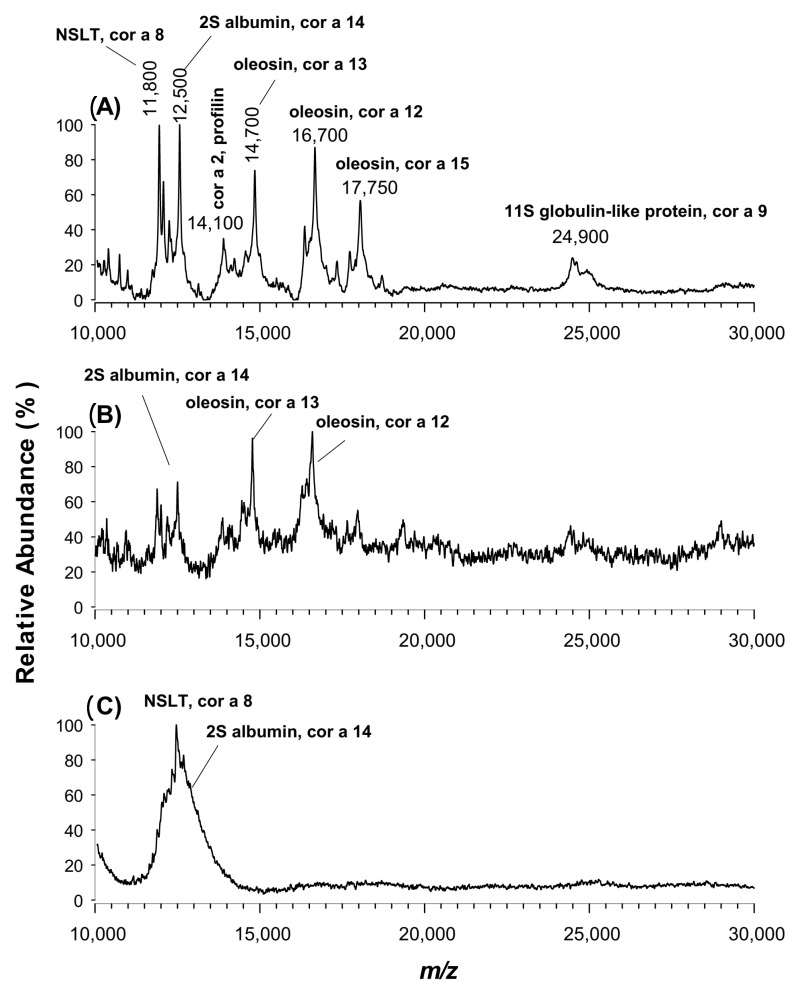
Positive MALDI-TOF MS spectra of the extract of hazelnut proteins mixed with (**A**) CPPA, (**B**) CHCA or (**C**) CClCA as the matrix. The identified proteins are labelled in the subfigures, NSLP (Non-specific lipid transfer protein).

Hazelnut (*Corylus avellana*) is among the most common tree nuts that induce food allergic reactions in sensitized people [51]. It is widely used in prepackaged pastry and ice cream products; traces of hazelnuts can accidentally be introduced into products across the whole food chain due to cross-contamination. The main allergenic proteins known in hazelnuts have been identified as Cor a 1 (17 kDa), Cor a 2 (14 kDa), Cor an 8 (12 kDa), Cor a 9 (globulin 11S), Cor an 11 (globulin 7S), a lipid transfer protein (LTP), two oleosin isoforms (Cor 12, Cor 13), an isoflavone reductase homologue (Cor 6), a luminal binding protein (Cor a10), a 2S albumin (Cor a 14) and a Cor a 9 isoform [52]. Even if the detection technique for allergen quantitation is liquid chromatography coupled to MS by electrospray ion source (LC-ESI-MS), which allows a sensitive multiplexed quantitation of allergen proteins, the use of MALDI-MS hold great interest due to the key advantage of rapidly screening a huge number of samples. Indeed, MALDI-MS can represent a promising alternative to antibody-based assays or LC-ESI-MS commonly employed in searching for declared or hidden allergen, owing to its ability to detect multiple proteins in complex matrices if a high sensitivity is guaranteed. To this aim, an aqueous extract of hazelnut proteins was prepared as described in the experimental section and analysed by MALDI-TOF MS using each of the novel CPPA (A), CHCA (B) and CClCA (C) matrices (see Figure 6). By comparing the experimental *m*/*z* values with literature data [52,53,54,55] and a dedicated database (https://www.anallergo.it/en/patients/allergens/tree-pollen/corylus-avellana.html), we were able to assign the main proteins occurring in a crude hazelnut extract. As can be ascertained from Figure 6, the use of CPPA allowed a more informative spectrum, with the detection of seven proteins compared to three ones observed using CHCA as a matrix; the use of CClCA caused a poorly resolved peak around 12 kDa. While the proteins Cor 8 (*m*/*z* 11,800), Cor 14 (*m*/*z* 12,500), Cor 2 (*m*/*z* 14,100), Cor 13 (*m*/*z* 14,700), Cor 12 (*m*/*z* 16,700), Cor a 15 (*m*/*z* 17,700) and Cor a 9 (*m*/*z* 24,900) were detectable with the CPPA matrix, only Cor 22, Cor a13 and Cor a12 were observed in the presence of CHCA as the matrix. No good signals were obtained using the SA matrix, which offered even worse results than CHCA (data not shown).

### 2.4. CPPA as a Matrix for Intact Bacterial Cells in MALDI-TOF MS

The key features of ease of use, rapidity, high throughput and low cost have permitted a formidable development of MALDI-TOF MS for the routine identification of microorganisms in clinical microbiology and diagnostic laboratories. Several studies have reported the capability of MALDI MS to replace and complement conventional techniques for the identification of bacterial and fungal strains, yeast isolates, filamentous fungi and dermatophytes, providing that detailed homogenization procedures are established. Two approaches have been proposed to characterize microorganisms: (i) mass spectra comparison with fingerprint database and (ii) matching of biomarker masses to a proteome database. In the first one, unique spectra generated from intact cells are compared with formerly collected fingerprint libraries commercially available today. This solution is simple, rapid and easily adjustable for routine use in diagnostic laboratories. Likewise, this method focuses in developing specific databases of unique and conserved peaks for species and subspecies identification, independently of the culture growth conditions [9]. In the second approach, the biomarker masses associated with an unidentified microorganism are recognized by matching experimental protein molecular masses with protein molecular masses predicted from sequenced genomes [56]. This method is based on the observation that many biomolecules detected between 4000 *m*/*z* and 15,000 *m*/*z* in MALDI-MS mass spectra of whole-cell extracts correspond to singly charged protonated proteins, as demonstrated by Ryzhov and Fenselau [57] and more recently by Momo et al. [58]. A systematic characterization of biomarkers performed on intact *E. coli* cells established that the biomolecules observed in MALDI-TOF mass spectra, namely abundant, basic, and medium-hydrophobicity ribosomal proteins from the inside of the bacterial cell [57], were efficiently ionized during the MALDI process [59]. Furthermore, the use of organic solvents and acidic conditions or acidic matrices facilitates the extraction of ribosomal proteins during the lysis of bacterial cells [60], and thus intact microorganisms can be directly processed without further pretreatment. The size and intensities of peaks depend on the matrix chosen; 2,5-dihydroxybenzoic acid (DHBA) and CHCA are optimal matrices for detecting lower-mass ions [61], with a detection of up to 10 kDa, while SA is preferred for ions with mass greater than 15 kDa [62], providing a lower sensitivity than CHCA.

**Figure 7:**
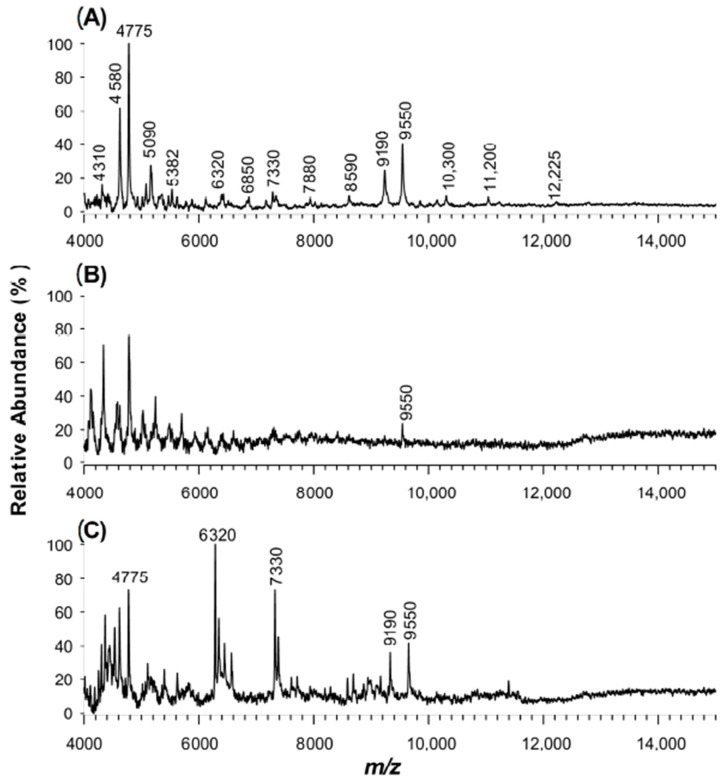
Positive MALDI-TOF MS spectra of *E. coli* cell culture (10^4^ cfu/mL) after mixing with (**A**) CPPA, (**B**) CHCA or (**C**) CClCA as the matrix. For signal attribution, see Table 1.

Recent studies have focused on determining the microbiological quality and the prevalence of potential human pathogenic bacteria occurring at low concentrations in lettuce salads, drinking water and surfaces in food markets [47]. Currently, the number of cultured cells needed to produce useful MALDI-MS reference spectra is around 10^8^–10^9^ cells/mL, but improved sensitivity is required. To investigate the capability of the CPPA matrix in bacterial profiling, we performed an acidic extraction of a diluted sample of *E. coli* (10^4^ cells/mL) and analysed the resulting solution by MALDI-TOF MS. For comparison, in Figure 7 are displayed the mass spectra using CPPA (A), CHCA (B) and CClCA (C). A higher number of significant peak signals was obtained in the case of CPPA and CClCA compared to CHCA, where a noisy uninformative spectrum was registered. Here, since the detected proteins exhibit relatively low MWs (i.e., <10 kDa), CClCA produced a good spectrum, confirming its efficacy in promoting the ionization of peptides or small proteins compared to CHCA [32]. Although it is not possible to distinguish a protein from its intact molecular weight, a tentative assignment of peaks detected in Figure 7A was pursued by combining literature data [58,63] with database mining. Specifically, the experimental *m*/*z* values were used as search parameters and were matched with sequence-derived theoretical MW values in the UniProtKB/Swiss-Prot database [58,64]. In Table 1, these results are summarized: 11 out of 15 of the matches corresponded to intracellular ribosomal proteins released after *E. coli* cell lysis. The experimental findings are fully consistent with the fact that ribosomal proteins are the most abundant proteins in *E. coli*, constituting up to 45% of the total mass of cells and up to 20% of the cells’ protein content [57,62].

Regarding MALDI-TOF mass spectra comparison, CPPA enabled obtaining a better protein fingerprint using lower laser energy and improved intra-spot and spot-to-spot repeatabilities, eliminating the laborious searching for ‘sweet’ points, with a resulting tenfold higher S/N ratio. Through this targeted approach, MALDI-MS can be used as a fast-analytical tool to generate protein profiling of different microorganisms, fungi and food compounds in a relatively short time and without significant sample pretreatment.

## 3. Materials and Methods

### 3.1. Materials

α-Cyano-4-hydroxycinnamic acid (CHCA), α-cyano-4-chlorocinnamic acid (CClCA), 3,5-dimethoxy-4-hydroxycinnamic acid (sinapinic acid, SA), ACTH 18-39 fragment, angiotensin I, renin, cytochrome *c* (horse heart), bovine myoglobin and trypsinogen were obtained from Sigma-Aldrich (Sigma Aldrich, St. Louis, MO, USA). Dimethyl sulphoxide (DMSO) used as solvent for NMR analysis was purchased from VARIAN. Water, acetonitrile (ACN), trifluoroacetic acid (TFAA), methanol (MeOH), tetrahydrofuran (THF) and chloroform (CHCl_3_) (Sigma Aldrich, St. Louis, MO, USA) were HPLC-grade and were used without further purification.

### 3.2. Chemical Synthesis of CPPA

CPPA was synthesized according to a standard Knoevenagel condensation reaction using cyanoacetic acid and cinnamaldehyde [32,33]. Ammonium acetate was used as a catalyst. Two grams of cyanoacetic acid (1 equiv.), 0.9 equiv. of the cinnamaldehyde and 0.15 equiv. of ammonium acetate were refluxed under stirring in sufficient amounts of toluene (ca. 50 mL). After quantitative separation of the reaction water by a Dean-Stark apparatus (ca. 3 h), the reaction mixture was cooled to 50 °C and filtered. The crude product was washed with enough amounts of distilled water and purified by recrystallization in 70% acetonitrile/30% water. A saturated solution of the reaction product was heated until boiling. When the solid was dissolved, the solution was cooled to room temperature and then on ice, since a precipitate was observed. Finally, the precipitate was filtered, and the procedure repeated thrice.

### 3.3. Matrix Characterization

A solution of CPPA (0.015 mM) was prepared in ACN:H_2_O (2:1) and the UV spectrum was acquired for both samples. For ^1^H-NMR analysis, CPPA was dissolved in deuterated DMSO (DMSO-d_6_) and a spectrum was registered. Laser desorption ionization analysis was carried out using a saturated solution of matrix in an ACN:H_2_O (2:1) mixture containing 0.1% TFA, which was spotted (1 µL) on the MALDI target plate.

### 3.4. Sample Preparations

#### 3.4.1. Standard Proteins

Stock solutions of 10 µM of each analyte in 0.1% TFA were prepared, diluted and mixed (1:1 *v*/*v* ratio) with the matrix prepared as above. Then, 1 µL of the sample was spotted directly on the target plate and analysed by MALDI-TOF MS. The same preparation was followed using CHCA (10 mg/mL in ACN:H_2_O, 2:1, 0.1% TFA), CClCA or SA as the matrix.

#### 3.4.2. Milk Proteins

One millilitre of bovine fresh milk was diluted in water (10 times), then 5 µL of the sample was mixed with CPPA or CHCA or SA (10 mg/mL in ACN:H_2_O (2:1)), and 1 µL was spotted directly on the target plate and analysed by MALDI-TOF MS.

#### 3.4.3. Hazelnut Proteins

First, the raw hazelnut samples were ground using a laboratory blender. To avoid sample overheating and to obtain good homogeneity, the grinding was carried out in 5 steps of 30 s each, interspersed with a 5 s pause by setting the speed to the maximum value. The ground product was finally sieved with a 2 mm sieve. The extraction procedure used was the following: 20 mL of extraction buffer 20 mM Tris-HCl pH 8.2 was added to 1 g of sample, mixed for 2 min, stirred at 26 °C for 1 h at a speed of 250 rpm, sonicated for 10 min and centrifuged for 15 min at 3000 rpm at a temperature of 18 °C. In this phase, the sample stratified into three phases: a bottom one consisting mostly of protein aggregates, a turbid central phase and a foamy surface phase constituted by the lipid component. The central phase was withdrawn, diluted 1:20 with ultra-pure water and filtered on 0.45 μm PTFE (polytetrafluoroethylene) syringe filters (13 mm diameter).

#### 3.4.4. Bacterial Proteins

*E. coli* (ATCC 25992) standard culture bacteria were obtained from LGC ATCC Standards (LGC Standards S.r.l., Milano, Italy). Glassware and media were subjected to autoclaving at 15 lbs of pressure for 15 min prior to bacterial culture. One colony of *E. coli* was carefully taken up from a freshly prepared agar plate using a sterile loop. The collected bacteria were cultured on nutrient broth (Oxoid) for 48 h at 42 °C up to a typical cell density of 10^10^ cells/mL. Serial dilutions were set up in saline solution (NaCl 0.9%) and 1 mL of the resulting dilution was inoculated into a nutrient medium (nutrient agar, Oxoid, Dublin, Ireland); the plates were then incubated at 42 °C for 24 h. After the incubation, the colonies were counted for each dilution, and the concentration of *E. coli* was deduced from the primary culture broth. To extract proteins, 500 µL of bacterial culture was centrifuged at 15,000 rpm for 5 min to pellet the cells. The supernatant was discarded, and the cells were washed twice with 1 mL of water. After each step, the cells were pelleted by centrifugation at 15,000 rpm for 5 min. The pellet was dissolved by vortexing for 2 min in 50–100 µL of 1.25% TFA.

### 3.5. Instrumentations

UV–visible spectra acquisition in the range 250–500 nm was performed using a UV1601 spectrophotometer (Shimadzu Italia S.r.l., Milan, Italy). ^1^H-NMR spectra were recorded with a Varian Unity Plus spectrometer (^1^H 400 MHz). Chemical shifts of ^1^H (δH) in parts per million were determined relative to DMSO-*d*_6_. A MALDI TOF/TOF 5800 system (AB SCIEX, Darmstadt, Germany) equipped with a neodymium-doped yttrium lithium fluoride (Nd:YLF) laser (345 nm), operating in linear positive ion mode, was used (typical mass accuracy was ≤100 ppm). At least 5000 laser shots were typically accumulated by a random rastering pattern, with a laser pulse rate of 400 Hz and a laser fluence of 1 mJ/cm^2^ in the MS mode. The delayed extraction (DE) time was set at 450 ns. Calibration in linear mode was carried out using a protein mixture composed of insulin beta chain (3497.0 Da), insulin (5735.0 Da) and cytochrome *c* (12361.1 Da).

### 3.6. Detailed Data Description and Computation Methods

DataExplorer 4.0 (AB Sciex, Darmstadt, Germany) was used to control data acquisitions and initial elaboration; SigmaPlot 14.5 was used to graph final mass spectra. ChemDraw Pro 8.0.3 (CambridgeSoft Corporation, Cambridge, MA, USA) was employed to draw chemical structures. Visualization and initial building were done by using ChemDraw Pro 10.0 and Chem3D Ultra 10.0. Initial geometries of the neutral and protonated compounds were created and optimized energetically by density functional theory (DFT) calculations. For proton affinity, DFTB3, based on a third-order expansion of the DFT total energy around a reference density, was used.

## 4. Conclusions

The α-cyano-5-phenyl-2,4-pentadienic acid (CPPA) matrix was synthetized and employed for intact proteins analysis by MALDI-TOF MS in positive ion mode. Unlike conventional organic matrices, CPPA showed a lower background signal, higher signal-to-noise ratio, and good spot-to-spot repeatability. Homogeneous spots provided by CPPA eliminated the laborious searching for ‘sweet’ points allowing qualitative determination. The successful performance of the CPPA matrix indicates its potential for protein detection in foodstuffs, which was demonstrated by the examination of cow milk and hazelnut samples. Most interesting is that CPPA was an efficient matrix for the analysis of intact microorganisms in clinical samples. Large-scale proteomic analysis by MALDI-MS using a CPPA matrix holds great promise.

## Figures and Tables

**Table 1:** Proteins identified after matching the experimental *m*/*z* values obtained in MALDI-TOF mass spectra of *E. coli* cell samples with selected *m*/*z* from literature [32,58] after a Swiss-Prot/TrEMBL database search.

Literature *m*/*z* Value	Experimental *m*/*z* Value	Identified Protein
4309.3	4310	*50S ribosomal protein L36*
4580.04	4580	*Osmotically inducible lipoprotein B*
-	4775 ([M + 2H]^2+^)	*30S ribosomal protein S16*
5095.33	5090	*Protein S22*
5380.55	5382	*50S ribosomal protein L34*
6314.92	6320	*50S ribosomal protein L32*
6855.88	6850	*Carbon storage regulator*
7332.3	7330	*Cold shock-like protein CspE*
7872.22	7880	*50S ribosomal protein L31*
-	8590	*50S ribosomal protein L2*
9190.21	9190	*30S ribosomal protein S16*
9553.44	9550	*30S ribosomal protein S20*
10,298.28	10,300	*50S ribosomal protein L25*
11,199.26	11,200	*50S ribosomal protein L23*
1227.40	12,225	*50S ribosomal protein L22*
